# Assembly of the Cardiac Pacemaking Complex: Electrogenic Principles of Sinoatrial Node Morphogenesis

**DOI:** 10.3390/jcdd8040040

**Published:** 2021-04-08

**Authors:** Marietta Easterling, Simone Rossi, Anthony J Mazzella, Michael Bressan

**Affiliations:** 1 Department of Cell Biology and Physiology, University of North Carolina at Chapel Hill, Chapel Hill, NC 27599, USA; m_easterling@unc.edu; 2 McAllister Heart Institute, University of North Carolina at Chapel Hill, Chapel Hill, NC 27599, USA; 3 Department of Mathematics, University of North Carolina, Chapel Hill, NC 27599, USA; srossi@email.unc.edu; 4 Division of Cardiology, University of North Carolina, Chapel Hill, NC 27599, USA; Anthony.Mazzella@unchealth.unc.edu

**Keywords:** sinoatrial node, cardiac pacemaker cell, morphogenesis, conduction

## Abstract

Cardiac pacemaker cells located in the sinoatrial node initiate the electrical impulses that drive rhythmic contraction of the heart. The sinoatrial node accounts for only a small proportion of the total mass of the heart yet must produce a stimulus of sufficient strength to stimulate the entire volume of downstream cardiac tissue. This requires balancing a delicate set of electrical interactions both within the sinoatrial node and with the downstream working myocardium. Understanding the fundamental features of these interactions is critical for defining vulnerabilities that arise in human arrhythmic disease and may provide insight towards the design and implementation of the next generation of potential cellular-based cardiac therapeutics. Here, we discuss physiological conditions that influence electrical impulse generation and propagation in the sinoatrial node and describe developmental events that construct the tissue-level architecture that appears necessary for sinoatrial node function.

## 1. Introduction

**Figure 1:**
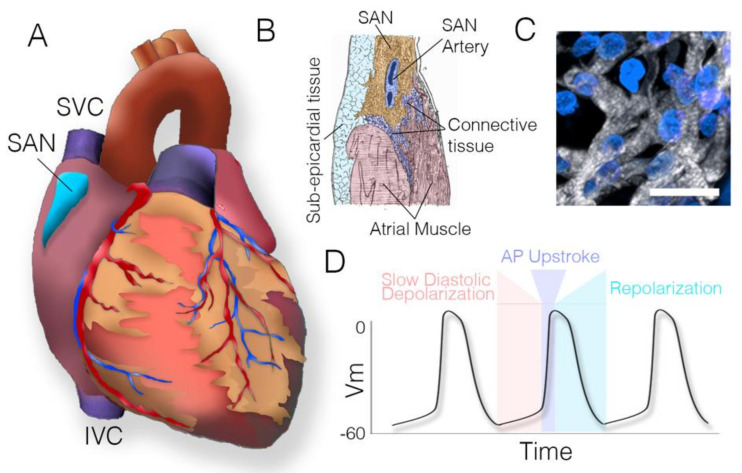
Anatomy and physiology of the sinoatrial node. (**A**) Diagram of the heart indicating the location of the sinoatrial node (blue). SAN—sinoatrial node, SVC—superior vena cava, IVC—inferior vena cava. (**B**) Sketch of sinoatrial node structure (modified from Keith and Flack 1907 [2]). Pacemaker myocardium (yellow) surrounds the sinoatrial node artery and is infiltrated and surrounded by connective tissue. (**C**) 30mm Z-stack through the sinoatrial node of an avian embryo. Pacemaker myocardium stained with MF20 (white) is arranged as loosely connected, unaligned fibers. Scale bar—20 mm. (**D**) Pacemaker cell action potential waveform. Vm—membrane potential.

The mature sinoatrial node is a complex, heterogeneous structure located at the junction of the right atrial myocardium and the superior vena cava. It extends posteriorly along the terminal crest towards the insertion of the inferior vena cava (Figure 1A–C) [1]. Since first being described by Keith and Flack in 1907 [2], the sinoatrial node has garnered a significant amount of interest based on its role as the primary pacemaking center of the heart. The sinoatrial node contains a unique class of cardiomyocytes termed cardiac pacemaker cells, which autonomously oscillate membrane potential leading to the rhythmic, spontaneous initiation of action potentials. Pacemaker cells display a wide variation in automaticity, which controls appropriate heart rate in response to various physiologic stimuli, primarily driven by sympathetic and parasympathetic inputs which modulate slow diastolic depolarization time (Figure 1D) [3,4]. Perturbations in sinoatrial node automaticity can be caused by acquired or inherited intrinsic cellular problems, or as the result of extrinsic stimuli such as exaggerated reflex responses or medication side effects. A pooled analysis of 20,572 patients from the Atherosclerosis Risk in Communities Study and the Cardiovascular Health Study reported the incidence of sinoatrial node dysfunction to be eight per 10,000 person-years. Increasing age, greater body mass index, hypertension and concomitant conduction disease were demonstrated to be associated with sinoatrial node dysfunction. Due to increases in these underlying factors, the incidence of sinoatrial node dysfunction is projected to double over the next 50 years [5].

Sinoatrial node dysfunction is a generalized term that represents a group of conditions, including inappropriate sinus bradycardia, sinus arrest, sinus exit block and tachycardia bradycardia syndromes. In addition to medication side effects and exaggerated vagal tone, degeneration of the sinoatrial node or surrounding atrial tissue can lead to sinoatrial node dysfunction by impeding impulse formation and impulse conduction. The gradual destruction of the sinoatrial node is generally associated with fibrotic remodeling and fatty infiltration leading to sclerodegeneration and eventual senescence [6]. Outside of primary sinoatrial degeneration, the destruction of the nodal-atrial interface or atrial wall can also lead to exit block or damage to the parasympathetic and sympathetic inputs to the sinoatrial node.

The clinical management of symptomatic sinoatrial dysfunction and inappropriate sinus bradycardia first focuses on alleviating secondary causes [7]. This includes removing stimuli that may depress sinoatrial node automaticity, such as exaggerated vagal tone or medications with adverse chronotropic effects. When this is not possible, a permanent transvenous pacemaker may be implanted. Although permanent atrial pacing is usually well-tolerated and associated with low procedural risk in experienced operators’ hands, it represents only a reactive treatment rather than a preventative therapy. Unfortunately, preventive therapies currently do not exist. For this reason, increasing emphasis is being placed on constructing a holistic understanding of sinoatrial node function to better predict the conditions that may lead to pathology and/or to formulate novel therapeutic approaches to correct sinoatrial node-related arhythmic disease. Towards this aim, a broad array of physiological [8,9,10], computational [11,12] and genetic studies [13,14,15,16] have built models of how the sinoatrial node’s unique cellular and molecular features impact cardiac pacemaker cell activity. The overriding goals of this review are to demonstrate how sinoatrial node morphogenesis creates a specialized microenvironmental niche that supports pacemaker cell function. Towards this aim we first describe some of the basic biophysical conditions that pacemaker cells natively encounter in the heart, outline models of how pacemaker cells are thought to overcome some of the functional barriers to which they are subjected and finally discuss events that occur during sinoatrial node morphogenesis that may serve to support the ability of cardiac pacemaker cells to generate and propagate electrical impulses.

## 2. Physiological Barriers to Sinoatrial Node Function/Source-Sink Relationships in the Sinoatrial Node

Estimations in humans suggest that approximately 10,000 pacemaker cells may reside in the central sinoatrial node [17] compared with ~2–4 billion working myocytes [18,19]. This fundamental numerical disadvantage places pacemaker cells in a very vulnerable position in which they serve as a small current source for the surrounding working myocardium. This configuration is antithetical to efficient electrical propagation. More specifically, for electrical impulses to successfully conduct through cardiac tissue, a few essential criteria must be met: each cell must be supplied with enough current to reach its depolarization threshold and generate a large enough current flow to activate downstream cells [20,21]. Under conditions in which few cells (such as pacemaker cells) are placed upstream of a large volume of electrically coupled tissue or a current sink (such as the working myocardium), the current generated by the small source rapidly dissipates across the sink before these cells can reach their threshold potential. Additionally, depending on the extent of electrical coupling and the size differential between the source and the sink, ionic gradients that form as the source begins to depolarize can activate local subthreshold current circuits which dampen the ability of the source to accumulate the charge needed to fire an action potential [21,22,23]. Theoretically, this would be predicted to prolong the time it takes for the source cells to reach threshold potential, delaying or potentially suppressing electrical impulse initiation. Under these conditions, a self-sustaining action potential cannot be achieved, resulting in propagation failure or block. Conduction failures of this form are often referred to as a source-sink mismatch. Based on the native source-sink relationships present between the sinoatrial node and working myocardium, the obvious question becomes how do the small numbers of pacemaker cells present in the heart drive cardiac activation [24]?

Indeed, dating back to the earliest computational models describing interactions between pacemaking cells and working myocardium, it has been appreciated that unique electro-anatomical features must be present to support the native activity of the sinoatrial node [24]. In a broad sense, we can describe the mechanisms by which pacemaker cells escape suppression as both increasing their efficiency to serve as a source and decreasing the hyperpolarizing load of the sink. As described below, several interrelated models have emerged that can be thought of as contributing to these parameters.

## 3. Electro-Anatomical Features That Support Sinoatrial Node Function

### 3.1. Synchronization

Cardiac pacemaker cells do not function in isolation and are often modeled as a system of coupled electrical oscillators [25,26,27,28]. As such, the dynamics of their network-level behaviors define the amount of ionic current that can be generated and serve as a source to adjacent tissue. Somewhat paradoxically, however, experimental observations have demonstrated that individual pacemaker cells have varying rates of activity, rhythmicity and current densities [29,30,31,32]. Thus, if considered as isolated units, each cell’s ability to autonomously oscillate charged ions would lead to complex and potentially detrimental electrical interactions. At any given time, different populations of pacemaker cells would be polarized, depolarizing or repolarizing, disrupting the required electrochemical gradients needed for action potentials to propagate in a coherent fashion and/or introducing potentially out-of-phase impulses leading to arrhythmia. To overcome this, networks of pacemaker cells synchronize to a common frequency [33] such that individual cells are at compatible states in their oscillatory phase throughout the process of electrical impulse generation. This increases the volume of cells available to serve as an electrical source for activating the adjacent working myocardium while also satisfying the basic conditions required for impulse propagation. Furthermore, evidence suggests that synchronization helps maintain the stability of biorhythmic activity. Work from Clay and Dehaan demonstrated that increasing the numbers of entrained spontaneously active cells decreases the potential influence of stoichiometric fluctuations in ion channel activity. Random electrical noise would be predicted to impact the rate and rhythmicity of individual cells significantly. However, this effect can be mitigated as more cells are added and entrained within the system [34]. Thus, synchronization is a critical functional characteristic of pacemaker cells, and the heart’s pacemaking complex can be thought of as collectives of thousands of cells activating in unison.

In determining how systems of oscillators entrain, the possible phase of each oscillator, the frequency variation within the population and the magnitude of information shared between units need to be considered [26,35,36]. In the context of pacemaker cells, these parameters would be most equivalent to variations in the waveform of the action potential, the offset of the action potential phase and the extent of electrical coupling between cells. Indeed, studies have shown that each of these features is interdependently modulated among pacemaker cells as they coalesce towards synchronization, with the strength of electrical coupling between cells appearing to be the primary driver of this process [37]. As each pacemaker independently oscillates, small amounts of current flow back and forth between them, altering the native periodicity of each cell and resulting in advancement towards a common frequency. Indeed, Anumonwo et al. demonstrated that the application of a brief external current can change the phase characteristics of individual pacemaker cells. This established that subthreshold electrical interactions among collections of pacemaker cells could alter the spontaneous cycle length of each cell in the system, driving populations of pacemaker cells towards mutual entrainment [38]. Furthermore, multiple studies have noted systems of discreet entrained behaviors emerge as coupling characteristics are altered among pacemaker cells. At low levels of coupling, pacemaker cells display independent oscillation. As coupling is progressively increased: (1) dynamic patterns of entrainment with varying frequency emerge, (2) 1:1 frequency entrainment with differing action potential waveforms is achieved and (3) finally, at relatively high coupling conductance, pacemaker cells display 1:1 frequency entrainment with matching waveforms. The absolute values of coupling needed to reach each behavioral regime vary slightly depending on the differences of the innate waveforms and periodicities that need to be overcome within the starting populations [39,40]. Thus, to achieve synchronization, cells with similar phase and frequency features require lower coupling than cells with more divergent starting characteristics.

It should be emphasized that synchronization denotes that the system of cells becomes entrained to a stable periodicity, not necessarily that all cells match the rate of any individual cell’s activity. Multiple studies have indicated that the rate at which systems of pacemaker cells synchronize is a complex function of the independent activities of the coupled cells and not merely a convergence to the cycle length of the fastest cell in the system [37,38,39,40,41,42,43]. For example, Mata et al. showed that the more heterogeneous cardiac pacemaker cells (CPCs), the shorter the cycle length at which they synchronize [43]. Furthermore, the process of synchronizing oscillatory rates does not necessarily imply that all cells in the system fire at the same time. Michaels et al. demonstrated that slight temporal differences in the phase of synchronized pacemaker cells can replicate the slow activation pattern typically associated with the sinoatrial node. In this study, the authors simulated fields of pacemaker cells in a two-dimensional array and noted synchronization occurred in a phase and coupling-dependent manner, as described above. Interestingly, propagating wavefronts were noted among the collections of synchronized cells. However, these wavefronts were not the direct result of action potential conduction but were due to discrepancies in the phase of the synchronized pacemaker cells. Thus, the slight offset in the activation times of synchronized cells gave rise to a region of central activation and a wave of depolarization that moved radially outward [44].

Collectively, studies related to the mechanisms of synchronization have thus far uncovered a trend towards uniform firing through a process of democratic mutual entrainment at sufficient coupling values. Additionally, these studies indicate that entrainment is not merely restricted to rates of activity but can also extend to the precise matching action potential waveform characteristics and give rise to intricate activation patterns. As such, the process of synchronization is critical for maximizing the number of cells that effectively serve as a source of charge for activating the downstream working myocardium.

A major challenge for studying synchronization of cardiac pacemaking activity is the multiscalar and heterogeneous nature of the sinoatrial node. Furthermore, the influence of the coupled atrial tissue is not typically considered when examining the process of synchronization. As described below, computational treatments of sinoatrial node function are continually adding increased complexity to our view of heterogeneity, including variations in gap junctional conductivities, ion channel conductance [45], cellular make up [46,47] and cell-to-cell connectivity [43]. In particular, recent models introduced by Gratz et al., Ly and Weinberg, Mata et al., and Sehgal et al. may be able to account for all these features in future analysis of synchronization [43,45,46,48].

### 3.2. Sinoatrial Node Structure—Interstitial Fibrosis

One of the defining histological characteristics of the mature sinoatrial node is the presence of large numbers of nonmuscle, fibroblast-like cells surrounding and interdigitated among the disorganized, unaligned pacemaker myocardium [49,50,51,52]. Studies have revealed the muscle/fibroblast ratio in the sinoatrial node varies between species, with mice displaying relatively low fibrotic content (~25% in adults) [53,54]. In comparison, pigs can show greater than 70% fibrotic content [49] while felines can display upwards of 90% fibrotic content in their sinoatrial node [55]. Furthermore, the fibrotic content of the sinoatrial node is not static and tends to increase with aging. Reports in humans indicate that fibrous connective tissue ranges from approximately 25–40% of the sinoatrial node in infants and children to greater than 70% in adulthood [55,56].

Despite the elevated connective tissue distribution in the sinoatrial node, mechanistic details outlining a direct relationship between fibrosis and pacemaker activity have remained challenging to demonstrate experimentally. Kohl et al. suggested that stretch-dependent activation of fibroblast ionic currents may raise the rate of pacemaker cell activity when these populations are electrically coupled [57]. Oren et al. proposed that fibroblasts may act as current sinks, conduction obstacles or establish independent conduction pathways supporting sinoatrial node activity. This work emphasized that the role of the fibroblast populations is largely dependent on their density, distribution, and the nature of their potential electronic interactions with pacemaker cells [58]. Karpaev et al. developed a computational model that considers the interaction between center and peripheral pacemaker cells, atrial cells and fibroblast interconnected via gap junctions [47]. In this work, it was determined that a sinoatrial node consisting of only myocytes fully activated in less than 8ms and failed to reproduce known central-to-peripheral propagation patterns observed in electrophysiological recordings of the sinoatrial node. By including fibroblasts electrically coupled with pacemaker cells, activation sequences consistent with those observed in vivo could be generated [47]. In support of this, studies have demonstrated that fibroblasts coupling can lead to the depolarization of myocytes [59,60], which, if extrapolated to pacemaker cells, might be predicted to protect against the hyperpolarization of the sinoatrial node. In additional studies, Miragoli et al. further demonstrated that myofibroblasts can induce spontaneous automaticity in otherwise senescent working myocytes, indicating that fibroblasts may support oscillatory behavior [61]. The above studies collectively imply direct electrical coupling between fibroblasts and pacemaker cells. There is some support for this idea in that lucifer yellow loading into sinoatrial node fibroblasts appeared to show spread into pacemaker myocardium [62]. Furthermore, cardiac fibroblasts express gap junctional proteins, including Connexin 43 and Connexin 45 [59,63]. However, if functional gap junctional coupling is present between pacemaker cells and fibroblasts, it would seem very rare within the sinoatrial node, as such junctions are not readily detected through ultrastructural analysis [64]. Collectively, the studies above indicate that sinoatrial node fibroblasts may play a role in modulating pacemaker cell excitability via cell-cell electrical interactions. However, further work is required to validate this idea in vivo.

Beyond a proposed direct role in the modulation of pacemaker cell electrical activity, sinoatrial node fibroblasts likely contribute to the sinoatrial node’s overall tissue-level biophysical properties. Herein, principles of discontinuous propagation would apply. Somewhat counterintuitively, this principle suggests the safety factor for propagation will increase as action potentials encounter regions of high electrical resistance [20]. For instance, work from Joyner et al. using numerical modeling demonstrated under the circumstance in which small numbers of cells must electrically drive large volumes of myocytes (such as the sinoatrial node/atrial boundary or the purkinje fiber/ventricular myocardial interface), resistive barriers can support propagation [65,66]. This theory was also demonstrated in work from Rohr et al. using cultured neonatal rat ventricular myocytes. Here it was shown that electrical propagation at sites of abrupt myocardial expansions (such as the sinoatrial node atrial boundary) was supported by discontinuities that lower cellular coupling [67]. As such, nonexcitable fibroblasts, and the extracellular matrix they deposit in the sinoatrial node, may function as resistive barriers that establish discontinuities in sinoatrial node conduction. This would support pacemaker cell excitability by preventing current dissipation during the slow diastolic and/or upstroke phase of pacemaker action potential generation. Thus, by creating a unique microenvironment, sinoatrial node fibrosis may provide direct biophysical support for pacemaker myocardium.

### 3.3. Sinoatrial Node Structure—Atrial Interface

A comprehensive review on sinoatrial node (SAN)/atrial coupling can be found in Joyner, Wilders and Wagner [11]. Below, we introduce some of the critical principles underlying this interaction. When the influence of the electrical load placed on the sinoatrial node by adjacent atrial tissue is modeled, the existence of three possible scenarios emerge: (1) no pacing, in which cardiac pacemaker cell electrical activity is entirely suppressed by the atrial myocardium, (2) pace but no drive, in which pacemaker cells can initiate an action potential but cannot stimulate the atrial tissue and (3) pace and drive, in which pacemaker cells generate an electrical impulse that successfully propagates to the atria [24,68]. In general, modeling has again highlighted coupling conductance within the sinoatrial node and/or between the sinoatrial node and the atria as the major determinants of successful propagation. When coupling conductance is large, the atrial cells hyperpolarize the sinoatrial node, and pacemaker cells are inhibited from reaching threshold membrane potentials, thus blocking action potential initiation (no pacing). When coupling conductance between the sinoatrial node and atria is too small, pacemaker cells display rhythmic activation but cannot transmit impulses to the atria (pace but no drive). Only for intermediate values of conductance can the sinoatrial node both sustain the capacity to fire an impulse and propagate it to the atria (pace and drive). Of note, the values of intermediate coupling for pacemaker cells in this case are still relatively low (2.6–7.5 nS) [69,70,71] when compared to the junctional conductance present in the atrial and ventricular working myocardium, which are between ~10–100 times higher [72,73]. Thus, it is generally accepted that the sinoatrial node must tightly regulate its level of coupling within a narrow range to sustain function. However, the mechanisms by which coupling is controlled on a macroscopic scale in and around pacemaker cells appear varied and complex [74].

**Figure 2:**
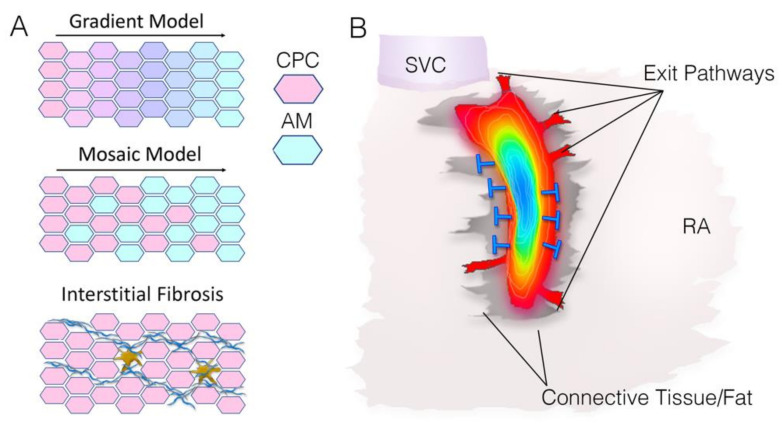
Models of sinoatrial node structure/function. (**A**) Graphical depictions of the Gradient Model, the Mosaic Model, and Interstitial Fibrosis. CPC—cardiac pacemaker cell, AM—atrial myocyte. (**B**) Diagram of sinoatrial node activation pattern (blue to red). Potential exit pathways to the atrial myocardium and connective tissue (grey) that serve as resistive barriers (blue lines) are indicated. SVC—superior vena cava, RA—right atrium.

Various models have been put forth to describe the possible means by which pacemaker cells mediate their electrical interactions with the atrial working myocardium. One such hypothesis suggests that there is a graded transition of phenotypic characteristics within the sinoatrial node. In this gradient model (Figure 2A) there is one distinct pacemaker cell type in the central sinoatrial node and a smooth transition in properties from the central sinoatrial node to its periphery [71,75]. Thus, characteristics such as ion current densities, coupling conductance and cell size vary through this continuum of phenotypes, allowing for ramping up properties such as junctional conductance (very low in the central sinoatrial node to relatively high at the periphery) [24,76]. An alternative model, known as the mosaic model (Figure 2A), postulates two populations of myocardial cells in the sinoatrial node (pacemaker and atrial cells), each with reasonably uniform properties (cell size and ionic currents, coupling, etc.). In this model, the percentage of each cell type in the various regions of the sinoatrial node dictates the localized electrophysiological properties. For example, junctional conductance would be relatively low in the central sinoatrial node because there are few atrial-like cells present. In contrast, these values increase towards the periphery because the percentage of atrial-like cells increases [39]. Although many authors have rejected the mosaic model based on comparison with experiments in rabbits [77], more recent computational simulations have used the mosaic model to elicit successful atrial pacing [78].

A still more elaborate description of the sinoatrial node/atrial boundary includes the existence of potential exit pathways (Figure 2B). This model is based on observations that the sinoatrial node in some species appears to be broadly surrounded by regions of fibrosis and fat with only a few discrete muscular connections linking pacemaker cells to atrial myocytes [79,80,81,82]. In theory, limiting the size of the interface between the sinoatrial node and atrial myocardium by having only a few discreet exit pathways would minimize the electrical load on the primary pacemaker cells, giving them sufficient time to build up the charge required to excite the atria [83]. In support of this, ablation of potential exit pathways has resulted in the sinoatrial node exit block [79]. High resolution functional and structural mapping of the adult sinoatrial node has lent support for this model [84,85] and three to five discrete branching myofiber tracts, located near the superior vena cava, interatrial septum and laterally atrial muscle, have been described [85,86]. Potential exit pathways are thought to be composed of transitional cells with morphological and functional characteristics between that of pacemaker and atrial cells [83]. While the existence of exit pathways has been documented in rabbit [71], dog [79,82] and human [84], several human studies have not found insulating fibrous barriers surrounding the sinoatrial node with defined exit sites [87,88,89,90]. In the work of Ho and Sánchez-Quintana, only some human hearts had a fibro-fatty tissue barrier between the node and the subendocardial myocardium [87]. Therefore, it has been suggested that exit sites may be functional, as opposed to anatomical features, of the sinoatrial node/atrial interface [91].

Computational studies have demonstrated that aspects of the gradient models, mosaic models and exit pathway models can each achieve functional pacemaking. Among the first mathematical investigations of the sinoatrial node/atrial interface was work conducted by Joyner and Capelle [24]. Using a radial description of the sinoatrial node, the authors were able to demonstrate that propagation from a central sinoatrial node region (with high intercellular resistance) to atrial tissue (with low intercellular resistance) was most efficient when a gradual transition in resistance was applied through the periphery of the sinoatrial node. Expanding on this, a study by Inada et al. modeled cells with graded phenotypic features (current densities, size, junctional conductance) from a central to a peripheral manner consistent with values recorded in the rabbit sinoatrial node [76]. This graded approach replicated critical features of sinoatrial node activation, including cycle length, activation and repolarization sequences and action potential waveforms. Using a mosaic model, which accounted for the geometric arrangement of pacemaker/atrial cells reported in Csepe et al. [84], Garza et al. were able to demonstrate that features consistent with graded phenotypes can emerge even in the absence of innate transitional phenotypes. However, this study does not account for observed phenotypic differences that have been noted among cells isolated from the sinoatrial node [78]. Kharche et al. generated a three-dimensional model of the sinoatrial node that considered a fibrotic border, a paranodal area and exit pathways [92]. To initiate electrical wave propagation close to the centroid of the 3D sinoatrial node, a gradient of the gap junctional conductance was required. Thus, a combination of the exit pathway and the gradient model were found effective in these 3D simulations. Collectively, these reports indicate multiple configurations of the sinoatrial node can support function. Indeed, variations in sinoatrial node architecture that have been noted across different species may suggest that one unified theory of a pacemaker cell/atrial interface may not be appropriate, and that elements of each of the above models may contribute to pacemaker function under different circumstances.

## 4. Sinoatrial Node Morphogenesis

One unfortunate aspect of the above models is that they are difficult to assay experimentally. For instance, altering conductance and/or modifying the fibrotic content specifically in the adult sinoatrial node is challenging. However, the increasing array of tools available to manipulate the heart during embryological development may be able to bridge some of the gaps between theory and application. For this to be effective, we require a precise spatiotemporal understanding of the micro and macroscopic embryonic assembly of the heart’s pacemaking complex. Therefore, in the following sections, we describe pacemaker cell development, highlighting studies that are starting to provide insight into the events underlying sinoatrial node morphogenesis.

### 4.1. Pacing in the Early Heart

During early cardiogenesis, the heart initially forms as a simple contractile tube. This tube displays innate electrical automaticity, as juvenile cardiomyocytes near the inflow segment of the tube spontaneously initiate action potentials that propagate towards the outflow [93,94,95]. However, the early pacing centers in the looping stage heart do not represent the progenitors of the mature sinoatrial node. Indeed, it appears that the location of the early pacing centers of the heart continuously shifts as the heart tube extends and elongates [95,96]. Direct labeling studies have confirmed that the pacemaking function is progressively assigned to new populations of myocytes as they are added to the venous pole of the heart. During these stages, bona fide sinoatrial node pacemaking cells reside as undifferentiated mesenchyme located posterior (in chick) or lateral (in mouse) to the primary heart tube [95,97,98]. The shifting of pacemaking activity continues until the late stages of looping morphogenesis, at which point myocardium located in the wall of the newly formed sinus venosus (along with the right inflow venous return of the heart) become electrically active, begin expressing functional markers of the pacemaker lineage and take on pacemaker cell functional characteristics [14,95,99,100,101,102,103,104,105,106]. These inflow myocardial cells go on to give rise to the pacemaker cells of the sinoatrial node [95].

Of note, fibrotic characteristics reminiscent of the mature sinoatrial node are not detected around the transient pacemaker populations during cardiac looping stages. These regions are structurally indistinguishable from their neighboring myocytes [94]. Despite this, the transient heart tube stage pacemaker centers still function. This functionality can be attributed, at least in part, to the electrophysiological properties of the early heart tube. For example, maximal diastolic depolarization in the linear heart has been reported at around −40mV, which increases to greater than −70mV by the completion of cardiac septation [107,108]. Therefore, the hyperpolarizing load of the heart tube in relation to the transient pacemaker centers is lower than in the more mature embryonic heart, which would be predicted to lessen source-sink burdens. Similarly, the conduction velocity in the heart tube is far lower than later stages or the adult heart [109]. While this can be partially explained by low sodium current (*I*_na_) in the younger heart [108], it is also likely due to the relative lack of intercellular gap junctional coupling. Indeed, the transcript levels of high conductance gap junctions, such as Connexin 40 and Connexin 43, are relatively low in the early heart tube [109]. Both transmission and freeze-fracture electron microscopy have indicated that early cardiomyocytes display gap junction to plasma membrane ratios far lower than their looping and septation stage counterparts [110,111]. Due to the lack of high conductance pathways between cells, the safety factor for propagation should be favorable for action potential initiation and propagation in the heart tube, as only a small fraction of the incoming depolarizing current would be lost to downstream cells.

Towards the end of looping morphogenesis (E9.5–E10 in mouse, E3 in chick), myocytes that will contribute to the mature sinoatrial node take over as the dominant pacemaker cells in the heart [95,97,99,100,104,112,113,114]. These newly differentiated pacemaker cells must drive a larger population of chamber myocardium than was present at earlier linear heart tube stages, and are confronted with electrogenic conditions that are less optimal. Specifically, maximal diastolic depolarization becomes progressively more negative during these stages as the working myocytes mature and gap junction expression and density increase [107,108,109,110,111]. Therefore, it would be expected that conditions present in the mature sinoatrial node thought to protect pacemaker cells from unfavorable electrical interactions with the adjacent working myocytes should manifest rapidly as pacemaker cells are added to the heart. Indeed, from the earliest stages of sinoatrial node pacemaker cell activity, these cells show lower transcript levels of Connexin 40 and Connexin 43 than the atrial myocardium to which they are connected [95,97,99,100,104,112,113,114]. Consequently, repression of gap junction transcription is believed to electrically insulate pacemaker cells right from the initial time point when they begin interacting with downstream atrial myocardium. However, the mature sinoatrial node’s structural features thought to help protect pacemaker function, including the presence of nonexcitable fibroblasts and higher extracellular matrix (ECM) content, are not present at these stages [104,115,116]. Instead, the tissue-level architecture of the sinoatrial node is built around the pacemaker myocardium after these cells have already taken on their physiological role of stimulating the downstream myocardium. It is with the rearrangement and the establishment of the surrounding microenvironment that features consistent with the mature sinoatrial node begin to arise.

### 4.2. Formation of the Pacemaker Cell Microenvironment

**Figure 3:**
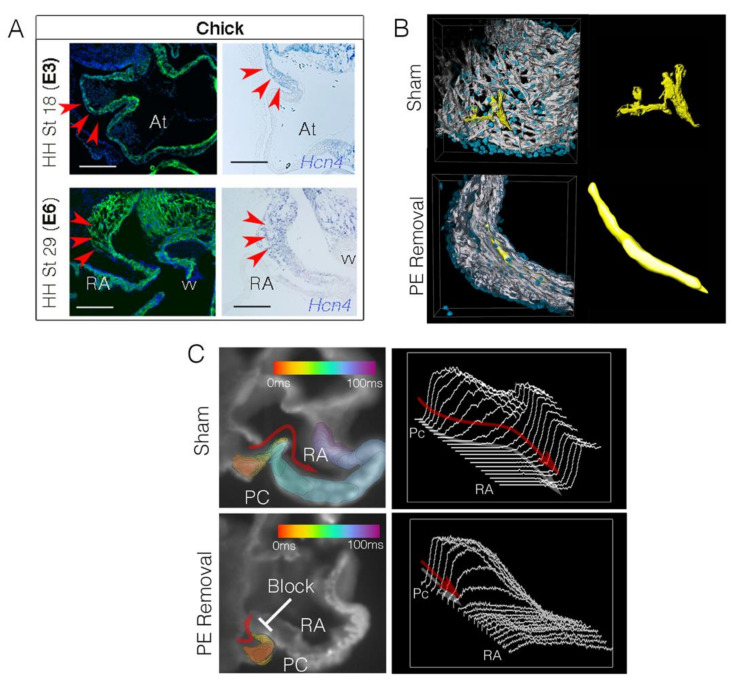
Embryonic remodeling of the sinoatrial node. (**A**) Sections through the chick sinoatrial node at Hamburger Hamilton (HH) stage 18 [121] equivalent to embryonic day 3 (E3) and HH stage 29 (E6). Sections are stained with muscle marker MF20 (green) and Dapi (blue). In situ hybridization for the pacemaker cell marker Hcn4 indicates the location of the sinoatrial node (red arrowheads) (reproduced from [105]). Scale bar–200 mm. (**B**) Volumetric reconstructions of the chick sinoatrial node at HH stage 29. Data are shown from a control embryo (Sham) and an embryo in which proepicardial cells have been blocked from entering the sinoatrial node. MF20−white, Dapi−blue. Groups of pacemaker cells have been reconstructed in yellow to demonstrate the change in morphology when proepicardial cells are prevented from entering the sinoatrial node. (**C**) Isochronal maps of impulse propagation through sections of the sinoatrial node/atrial junction. Following proepicardial removal, sinoatrial node conduction block occurs (reproduced with permission from [104]). PC-pacemaker cells, RA-right atrium.

Despite the variety of potential physiological roles that fibroblasts may play in pacemaking function, and the apparent conservation of their presence in the mammalian and avian sinoatrial node, the developmental patterning events that lead to the unique density of these cells found around pacemaker cells have only begun to be investigated. As described above, during the earliest stages of pacemaker cell activation in the embryonic heart, fibroblast-like cells are not detectable among pacemaker myocardium. However, it has recently been demonstrated that the tissue-level architecture of the forming sinoatrial node rapidly changes over cardiac developmental stages following pacemaker cell differentiation. During this period, the forming sinoatrial node remodels from a thin walled, densely packed collection of pacemaker myocardium, into loosely connected clusters or strands of cells surrounded by nonmuscle mesenchymal cells (Figure 3A). This functional reorganization occurs over an approximately three-day developmental window in avian (spanning E3–E6) [104]. Notably, the tissue-level morphological changes observed in the forming avian sinoatrial node seem to be preserved as they can also be tracked in mice [104].

Remodeling of the sinoatrial node region is driven, at least in part, by the integration of cells derived from an adjacent structure known as the proepicardium [104]. The proepicardium is a transitory, multipotent, embryonic tissue that forms along the anterior, ventral aspect of the junction between the liver bud and the inflow myocardium in which pacemaker cells reside [117,118,119,120]. Classically, cells from the proepicardium are described as extending and/or budding into the pericardial space between the liver bud and the atrioventricular myocardium. Proepicardial cells then bind to and spread over the myocardium giving rise to the epicardial layer of the heart. Subsequently, proepicardial-derived cells invade the myocardium contributing to cardiac lineages including fibroblasts, vascular endothelium and coronary smooth muscle [118]. Cell tracing studies in both chick and mouse have demonstrated that in addition to this traditional view of proepicardial migration, proepicardial-derived cells also rapidly and specifically invade the pacemaker myocardium at the end of looping morphogenesis [104]. Indeed, these cells can be seen wrapping around pacemaker cells, physically segregating them into unaligned, loosely connected clusters [104].

The incorporation of proepicardial cells into the sinoatrial node and their effect on pacemaker cell distribution creates a tissue architecture very analogous to the patchy fibrosis noted during the pathological remodeling of the ventricular myocardium. When viewed from the perspective of ventricular electrophysiology, creating regions of patchy fibrosis disrupts the three-dimensional syncytial nature of the myocardium. As reviewed by Nguyen et al., this can be modeled as creating interacting tangles of quasi-1D myocardial cables [122]. By adjusting the electrical connectivity of these cables from a 3D syncytium to 1D cables, source-sink relationships and the ability of the surrounding tissue to suppress spontaneous electrical activity decreases. Indeed, estimates for the number of cells required to generate enough current to stimulate an ectopic action potential in healthy 3D ventricular myocardium is on the order of 700,000 myocytes firing in synchrony [123,124]. However, by altering the tissue structure through fibrotic remodeling, this estimate drops by several orders of magnitude [122,124]. Patchy fibrosis is viewed as a significant predisposing factor to arrhythmogenic potential in the ventricles. However, when viewed from the perspective of pacemaker cell function, this form of fibrotic remodeling would be beneficial, lowering the number of pacemaker cells required to serve as a current source for the rest of the heart. In support of this, blocking proepicardial cell invasion into the forming sinoatrial node prevents structural remodeling leading to electrical failure (Figure 3B,C). Following proepicardial removal, pacemaker cells condense into aligned, densely packed muscle fibers reminiscent of the atrial chamber myocardium (Figure 3B). In this configuration, pacemaker cells display a drop in their ability to rhythmically initiate action potentials and demonstrate a failure to stimulate the adjacent atria (Figure 3C). Retrograde atrial-to pacemaker conduction is preserved, indicating that electrical connections are maintained following proepicardial removal [104]. However, the sinoatrial node cannot activate the larger volume of atrial myocardium to which it was coupled. Of note, the electrophysiological defects observed in hearts following the blocking of embryonic pacemaker cell/fibroblast integration are consistent with computational simulations in which propagation towards increasing electrical load without resistive barriers was conducted [65,66,125], and match closely to the results of Rohr et al. tracking electrical propagation through regions of tissue expansion [67,126]. These data suggest that the integration of fibroblasts into the sinoatrial node interstitium is critical for balancing source-sink relationships as cardiac morphogenesis proceeds and the electrical load that downstream myocardial cells place on pacemaker cells becomes untenable.

In addition to remodeling of the sinoatrial node interstitium, there is a clear heterogeneity in the embryonic sinoatrial node with regard to molecular identity during these stages of development. For instance, the embryonic sinoatrial node has a head region which is positive for the transcription factors *Tbx18*, *Tbx3*, *Shox2*, *Isl1* and negative for the transcription factor *Nkx2.5* [99,103,113,114]. These factors set up a transcriptional landscape believed to drive the pacemaker cell genetic program [13,15,16,17]. Along the tail region of the embryonic sinoatrial node, which serves as a junction to the atrial chamber myocardium, a slightly different genetic program appears to be in place. This region expresses many of the makers of the sinoatrial node head but is also positive for Nkx2.5. By manipulating transcription factor expression directly in this sinoatrial node/atrial junction region, Ye et al. and Li et al. were able to demonstrate this region is necessary to maintain proper sinoatrial node/atrial electrical interactions [102,127]. As such, the peripheral tail cells along the developing sinoatrial node/atrial interface may represent a transitional cell with phenotypic characteristics in between those of pacemaker cells and atrial myocytes.

### 4.3. Signaling Environment of the Embryonic Sinoatrial Node

The events that recruit fibroblast-like cell incorporation into the embryonic sinoatrial node, and/or organize the boundaries between the cell types associated with this region of the heart (head, tail, atrial chamber myocardium), are not fully understood. Related to this first point, while it has yet to be experimentally determined how fibroblast-like cells are recruited to the developing sinoatrial node, there is a large amount of data that may allow us to speculate. Indeed, the developmental window in which proepicardial cells invade the forming sinoatrial node has been examined by several groups using next-generation sequencing approaches. These studies highlighted a core signaling program in the embryonic sinoatrial node that seems likely to contribute to sinoatrial node morphogenesis. Vedantham et al. used laser capture microscopy followed by RNA sequencing (RNAseq) to compare differentially expressed genes between the embryonic sinoatrial node and atrial myocardium. Among the enriched genes noted in this study were members of the TGFb/BMP paracrine axis and known downstream targets of TGFb and BMP signaling [128]. While it should be noted that several paracrine factors were shown to be enriched in this report, the TGFb/BMP pathway is worth mentioning for several reasons that will be elaborated on below.

In sequencing studies conducted by Liang et al. and Van Eif et al., the enrichment of both upstream and downstream mediators of TGFb/BMP signaling was also demonstrated [114,129]. In these studies, sinoatrial node cells were sorted from transgenic animals carrying various fluorescence reporters in the pacemaker cell lineage (Hcn4-CreERT2 Isl1^fl/fl^, or Tbx3^+Venus^ respectively [114,129]), and these cells were processed for bulk RNA sequencing. Indeed, based on their findings, Van Eif et al. directly examined TGFb/BMP activity in forming the sinoatrial node and noted nuclear localization of phosoSMAD1/5/8 [129]. This data indicated that TGFb/BMP signaling is active in this region. Furthermore, the Van Eif study confirmed that BMP2 could induce the expression of a subset of pacemaker cell genes. More recent Single-cell RNA sequencing studies by Li et al. and Goodyer et al. further confirmed the enrichment of the TGFb/BMP signaling pathway in the embryonic sinoatrial node myocytes [127,130]. In these studies, single cells from the embryonic sinoatrial node (mouse 12.5 and E17.5) were sequenced, allowing for subpopulations to be identified within the heterogeneous cell populations of the sinoatrial node.

The above sequencing studies have spanned a range of embryonic stages from E12.5–E17 in mouse, a developmental window during which proepicardial cells actively integrate with pacemaker myocardium [104]. Significantly, TGFb/BMP pathway enrichment has been noted at earlier time points as well. Examining the transcriptional profile of the forming sinoatrial node just before structural remodeling indicated that BMP2, BMP4, and TGFB2 were all upregulated compared to the atrial myocardium, indicating these factors may contribute to the initial recruitment of proepicardial cells [104]. In adults, these factors are continued to be expressed in the SAN. Linscheid et al. and Brennan et al. noted the persistent enrichment of TGFb/BMP signaling factors in the adult sinoatrial node [131,132]. Therefore, the TGFb/BMP signaling is well positioned throughout life to influence sinoatrial node activity.

### 4.4. Potential Roles for TGFb/BMP Signaling in Sinoatrial Node Morphogenesis

While the functional role of BMPs and TGFbs has been extensively studied in the heart and is the subject of several excellent reviews (examples include [133,134,135,136,137,138,139]), we highlight some features of this pathway that may suggest their potential function in sinoatrial node morphogenesis.

**Figure 4:**
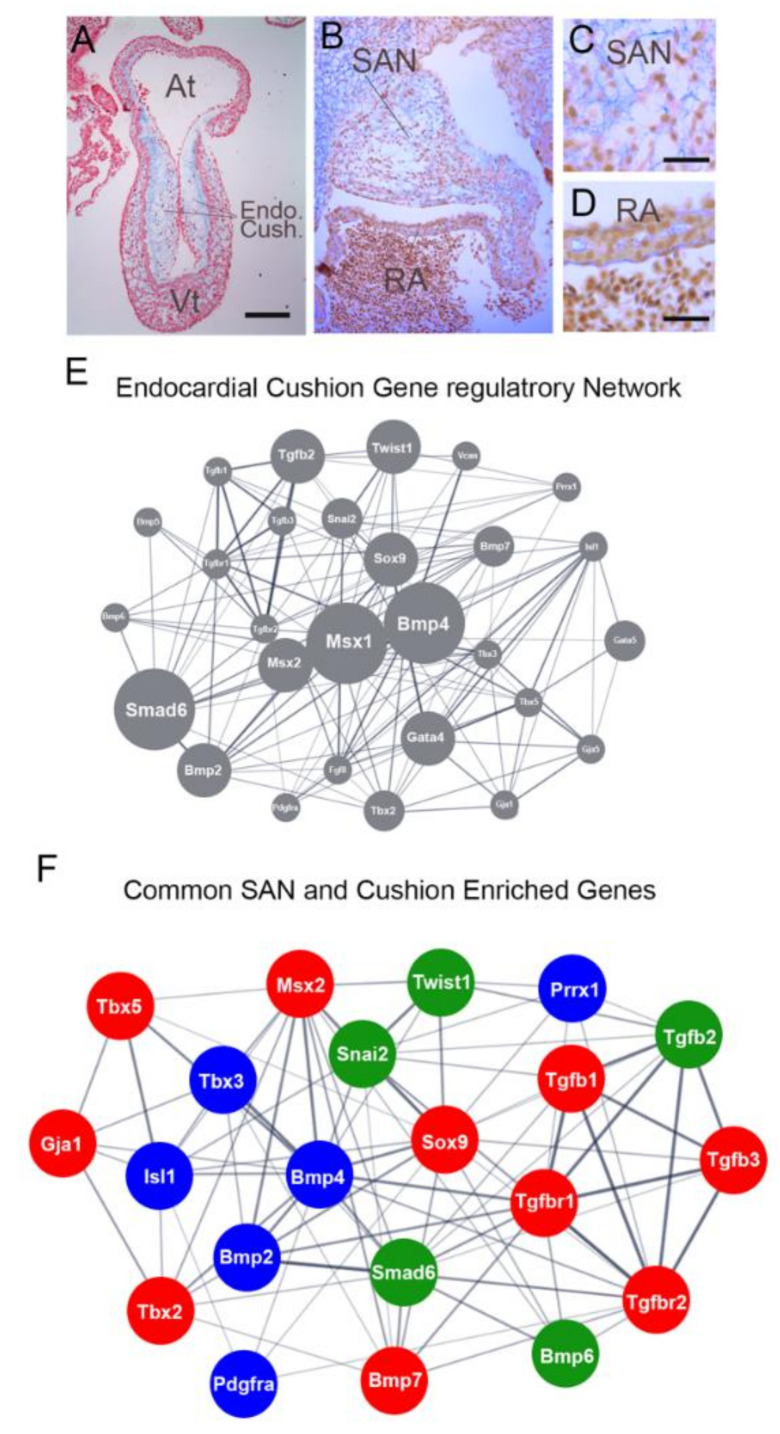
Genetic pathways active during sinoatrial node remodeling. (**A**) Sagittal section through an HH stage 19 chick heart stained with alcian blue (blue) which labels HA and nuclear fast red (red). Endocardial cushions are indicated. Scale bare—500 mm. (**B**) Section through the remodeling sinoatrial node of a chick embryo at HH stage 29. Sinoatrial node (SAN) and right atrium (RA are indicated). (**C**,**D**) Higher magnification images from (**B**). Scale Bars—50 mm. (**E**) Genes list was generated by comparing the Gene Ontology (GO) term Endocardial Cushion Development (0003197) with the gene regulatory networks published by DeLaughter et al. [149] and enriched genes from Schroeder et al. [150] and Singh et al. [151]. Node size reflects the number of data sets in which the indicated gene is present. Line thickness denotes confidence of potential interaction between genes [152]. (**F**) Comparison of genes identified as upregulated in the sinoatrial node based on sequencing studies from Vedantham et al., Bressan et al., and Van Eif et al. [104,128,129] Genes with greater than two-fold enrichment over atrial myocardium in all three data sets were compared to the gene-regulatory network for cushion formation in (**D**). Genes in blue nodes were enriched in all three RNA sequencing data sets, green nodes were present in two data sets, red nodes were detected in one data set.

BMP2/4/6/7, and TGFB1/2 and TGFB receptors2/3 have been identified as upstream factors that induce the morphological process of endocardial cushion formation in the atrioventricular canal and outflow components of the heart [140,141]. Here, a proteoglycan and hyaluronan-rich extracellular matrix termed the cardiac jelly is deposited and maintained in the basement membrane between the endocardium and myocardium (Figure 4A). This causes a swelling or separation between endocardial and myocardial layers of the heart that is, at first, acellular but becomes progressively populated by both endocardial and epicardial derived cells [133,142]. The combination of BMPs and TGFBs activates various cellular behaviors during cushion morphogenesis, including ECM deposition and endocardial cell invasion into the cushion structures [137,138,139]. While no cushions form near the embryonic sinoatrial node (Figure 4B,C), downstream effectors of TGFb/BMP signaling involved in cushion morphogenesis are enriched in several of the above-referenced sinoatrial node sequencing studies (Figure 4E,F) [104,125,126]. This overlap may suggest a process analogous to cushion formation is occurring during sinoatrial node formation. However, instead of generating an acellular cardiac jelly, it results in the remodeling of the pacemaker interstitium and transitioning this region into the patchy myocardium alluded to above.

The second point worth discussing relates to the known roles of TGFb/BMP in directing proepicardial derived cell behavior. Ishii et al. demonstrated that primary proepicardial cells would orient their axis of migration specifically towards sources of BMP2/4. While this study was focused on the paracrine factors that induce proepicardial outgrowth towards the embryonic atrioventricular junction, the shared enrichment of BMP2/4 in the forming sinoatrial node would highlight these factors as potential drivers of proepicardial cell recruitment into the pacemaking region of the heart [143]. Hill et al. further showed that BMP2 treatment results in loss of epithelial character and decreased cell-cell adhesion in an immortalized epicardial cell line [144]. These studies indicated that the expression of BMP2/4 by pacemaker cells would prime adjacent proepicardial/epicardial cells towards an invasive phenotype. Furthermore, Craig et al. demonstrated that TGFB2 is known to induce Hyaluronan synthase 2 (Has2) expression in epicardial cells [145]. High molecular weight hyaluronan, combined with TGFB2, stimulates epicardial cell epithelial-to-mesenchymal transition (EMT) and cellular invasion. Of note, the remodeling embryonic sinoatrial node displays high interstitial HA levels (Figure 4B,C), and Has2 is elevated in several of the reported sequencing studies referenced above [104,128]. Thus, the paracrine microenvironment that emerges during sinoatrial node development does appear consistent with epicardial cell recruitment and invasion. Additionally, among the factors that BMP2/4 activated in the epicardial cells is elastin (ELN) [146]. This is of particular note as the elastic nature of the sinoatrial node extracellular matrix has been noted by several groups, and ELN is deposited at high levels in the interstitial spaces of the mature sinoatrial node [131,147,148].

Finally, there is a complex interplay between TGFbs and BMPs during the process of myofibroblasts activation. TGFbs (typically TGFb1) are generally associated with the fibroblast-to myofibroblast transition that often occurs during wound healing and repair. During these processes, fibroblasts upregulate alpha-smooth muscle actin, become proliferative, and take on a contractile/synthetic phenotype in which they produce high levels of collagens, fibronectin and matrix metalloproteinases [152,153,154]. As a result, activated myofibroblasts are primed to generate and remodel extracellular microenvironments. However, it should be noted that BMP signaling appears to dampen or attenuate TGFb-mediated fibrotic remodeling in the heart [155,156,157]. Thus, how these signaling pathways interact during sinoatrial node morphogenesis requires further exploration.

## 5. Conclusions and Future Perspectives

Sinoatrial node dysfunction represents a significant clinical burden in the US, accounting for the surgical implantation of ~200,000 pacing devices each year [158] and over one million pacing devices worldwide [159]. Due to challenges associated with the implantation of pacing devices (particularly in young patients), the creation of tissue-engineered pacemaker cells to combat arrhythmic diseases has gained general interest over the past 20 years [17,160,161,162,163,164,165,166,167]. Significant effort is now being placed on exploring cellular-based therapeutics for correcting cardiac pacemaker cell dysfunction. However, the design and implementation of the next generation of cellular therapeutics will require a detailed understanding of the functional roles of all of the cell types in the sinoatrial node as well as an appreciation of the electronic interactions that govern sinoatrial node function. Here, we have described the physiological barriers pacemaker cells face, the mechanisms by which these barriers may be overcome, and the developmental events that lead to the construction of a competent sinoatrial node. Big questions remain, mainly related to applying theoretical understandings of pacemaker cell activity to the cellular and molecular regulators of sinoatrial node architecture. For instance, how does sinoatrial node developmental morphogenesis influence the parameters that dictate synchronization and what direct roles do BMPs and TGFbs play in sinoatrial node patterning?

Addressing this intersection between tissue-level sinoatrial node structure and function is of significant clinical interest, to establish a framework through which the vulnerabilities leading to pacemaker dysfunction can be viewed. Moreover, developing a holistic model of sinoatrial node electro-anatomy can provide a template on which future cellular-based therapeutics can be built. Herein, examining the process of sinoatrial node self-assembly during embryonic development can serve as a foundational resource for advancing our basic scientific and clinical understanding of cardiac pacemaking and inspire the next generation of interventional approaches.
